# Associations Between Different Dietary Vitamins and the Risk of Obesity in Children and Adolescents: A Machine Learning Approach

**DOI:** 10.3389/fendo.2021.816975

**Published:** 2022-02-17

**Authors:** Weifeng Tang, Wenqiang Zhan, Mengdan Wei, Qian Chen

**Affiliations:** ^1^ Ministry of Education-Shanghai Key Laboratory of Children’s Environmental Health, Xinhua Hospital, Shanghai Jiao Tong University School of Medicine, Shanghai, China; ^2^ School of Public Health, Shanghai Jiao Tong University School of Medicine, Shanghai, China

**Keywords:** multivitamins, children, adolescents, obesity, Bayesian Kernel machine regressioN, diet

## Abstract

**Backgrounds:**

Simultaneous dietary intake of vitamins is considered as a common and real scenario in daily life. However, limited prospective studies have evaluated the association between multivitamins intake and obesity in children and adolescents.

**Objectives:**

This study aimed to evaluate the relationship between the intake of different dietary vitamins and the risk of obesity in children (6-11 years) and adolescents (12-19 years).

**Methods:**

We conducted a cross-sectional study based on data from U.S. National Health and Nutrition Examination Survey, 2013-2016. A total of 3634 children and adolescents were included who had available data on dietary vitamins, obesity and covariates. We analyzed the dietary intake levels of nine vitamins, including vitamin A, vitamin B_1_, vitamin B_2_, vitamin B_6_, vitamin B_12_, vitamin C, vitamin D, vitamin E, vitamin K. Multivariate logistic regression was used to model the associations between vitamins and obesity. Bayesian kernel machine regression (BKMR) was employed to explore the joint and independent effects of vitamins on obesity among children and adolescents.

**Results:**

In the multivariate logistic regression model, five vitamins (vitamin A, vitamin B_1_, vitamin B_2_, vitamin B_12_, and vitamin D) were negatively associated with obesity in children and adolescents. BKMR analysis showed that when the concentration of the nine vitamins was at or above the 55th percentile compared with the median value, the combined intake of these vitamins could significantly reduce the risk of obesity in children and adolescents. Potential interactions between vitamin B_2_ and vitamin B_12_ in increasing the risk of obesity in children and adolescents were observed.

**Conclusions:**

We determine the combined effects of multivitamins on obesity in children and adolescents, and observe a significant interaction between vitamin B_2_ and vitamin B_12_. Further cohort studies are needed to clarify the health effects of multivitamins intake in a larger population.

## Introduction

Obesity is a complex disease, which is intertwined with biological, developmental, environmental, behavioral and genetic factors (1). Given that the obesity rate of children and adolescents worldwide is rising, it is necessary to pay special attention to the obesity in children and adolescents (2). Childhood obesity is a continuing and serious international health problem affecting approximately 17% of American children and adolescents, and will threaten their health and longevity in adulthood (3). However, the reasons for the large differences in the prevalence of obesity among children are still unclear.

Childhood is identified as the golden age of investing in obesity prevention. Considering the multi-factor origin of obesity, a better understanding of the influencing factors is essential for effective treatment and prevention (4, 5). Micronutrient status may be a significant factor for the development of obesity during childhood (6). At the same time, due to the high correlation between dietary intake of vitamins, studies had shown that there was a negative correlation between vitamin A, vitamin B_12_ and folic acid status and the intake of thiamine and riboflavin (7, 8). Therefore, it is urgent to examine the significant effects of common multivitamins in children and adolescents on obesity. Most vitamins are deficient in obese individuals, especially water-soluble vitamins, such as vitamin B_12_ and vitamin C. However, in the case of obesity, multivitamins are less evaluated. Adipose tissue is considered to be a metabolic and endocrine organ (9). Excessive intakes of vitamins can interfere with body homeostasis, but vitamins deficiency can exacerbate pathological conditions (10). Therefore, there is a great need to assess the relationship between vitamins status and obesity in children and adolescent.

However, the majority of the current nutritional epidemiological studies only evaluated the impact of individual vitamin intake on childhood obesity (11–14). Those studies only focused on the association between vitamins in isolation and childhood obesity, rather than considering all vitamins intakes, which might have additive, synergistic, antagonistic, or enhancing effects when they took together. In view of the urgent need for new methods, the development of multiple exposure model statistical methods has recently received considerable attention (15). However, few studies had taken multivitamins as a whole and used advanced statistical methods to examine vitamin mixtures (16). Bayesian Kernel Machine Regression (BKMR) is a novel statistical method that solves interactions and nonlinear relationships by flexibly modeling exposures, and has been widely used in many different medical research fields (17, 18).

Therefore, this study aims to explore the association between 9 vitamins and obesity in children and adolescents aged 6 to 19 in the U.S. National Health and Nutrition Examination Survey, including vitamin A, vitamin B_1_, vitamin B_2_, vitamin B_6_, vitamin B_12_, vitamin C, vitamin D, vitamin E, vitamin K. Given the effect of multivitamins intake, we use BKMR model to examine the joint effect of multivitamins on obesity in children and adolescents and assess the interaction between vitamins.

## Methods

### Study Participants

**Figure 1:**
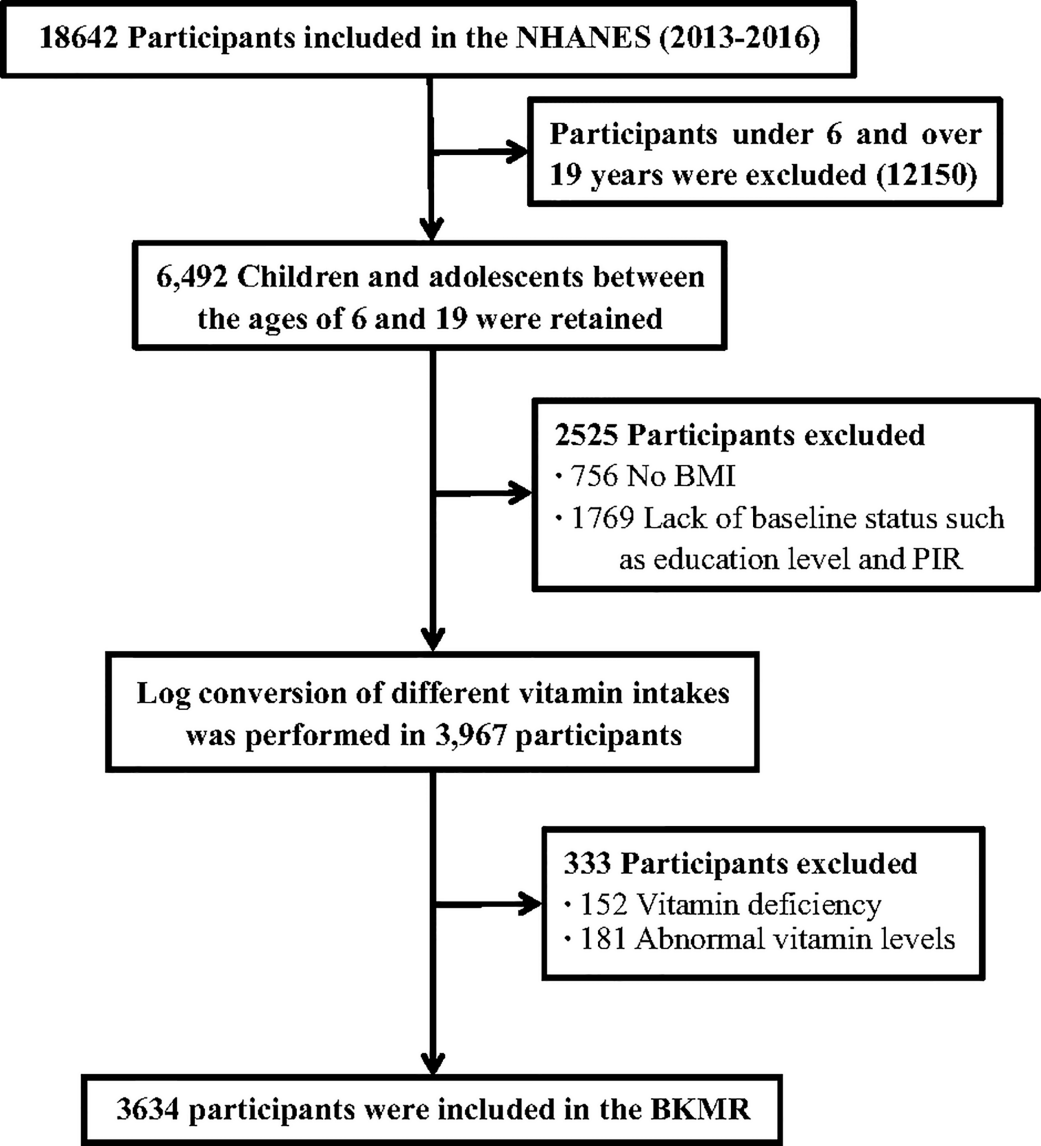
Selection process of subjects. BKMR, Bayesian kernel machine regression.

The current study used cross-sectional data from National Health and Nutrition Examination Survey (NHANES) 2013-2016. NHANES is a survey designed to assess the health and nutrition of a nationally representative sample of the non-institutionalized U.S. population. A detailed description of the study design has been previously published (19). The survey is maintained and managed by the National Bureau of Statistics of the Center for Health Statistics (NCHS) and the Centers for Disease Control and Prevention (CDC). Participants aged 6-19 years with complete dietary intake data were included in the study population. Participants with unreliable diet data, missing vitamin data, or potential confounding factors were further excluded from the analysis, and a total of 3634 subjects were included in the analysis. The detailed flow chart of this study was shown in **Figure 1**. All participants provided written informed consent and the Institutional Review Board of the NCHS approved the survey protocol (20).

### Exposure and Outcome Variables

NHANES used 24-hour dietary recalls (24HRs) to obtain dietary information. For each participant, daily intake of nutrients from food/beverages and dietary supplements was calculated using the US Department of Agriculture’s Diet and Nutrition Database for dietary research and NHANES dietary supplement calculations, respectively. The intake level of nine common vitamins (vitamin A, vitamin B_1_, vitamin B_2_, vitamin B_6_, vitamin B_12_, vitamin C, vitamin D, vitamin E, vitamin K) of each participant was assessed by the food frequency questionnaire. During the health check in the mobile check center, the weight and height were collected by well-trained health technicians. Body mass index (BMI) category was calculated for children and adolescents aged 6–19 years in NHANES and defined as four levels: underweight (BMI < 5th percentile), normal weight (5th ≤ BMI < 85th percentile), overweight (85th ≤ BMI < 95th percentile) and obese (BMI ≥ 95th percentile) (21).

### Covariates

Demographic characteristics were obtained through questionnaire surveys, including age (years), ratio of family income to poverty (PIR), energy, and serum cotinine concentration as continuous variables. Education level was grouped into high school and above, high school, elementary school and below. The race was divided into Mexican American, Other Hispanic, Non-Hispanic White, Non-Hispanic Black, Other/multi-racial (22).

According to National Cholesterol Education Program guidelines (23), abnormal serum TC, serum HDL-C, serum LDL-C, fasting TAG and fasting plasma glucose concentrations, and abnormal HOMA-IR score, were defined as follows: TC ≥ 200 mg/dl, HDL-C ≤ 35 mg/dl, LDL-C ≥ 130 mg/dl, TAG ≥ 150 mg/dl, glucose ≥ 100 mg/dl and HOMA-IR ≥ 4.39 (24).

### Statistical Analysis

Continuous variables were expressed as mean ± standard deviation or median [interquartile range (IQR)], and the number of categorical variables (percentage) was expressed. Spearman correlation was used to evaluate the association between dietary vitamin intake and obesity in children and adolescents. Given that the distributions of vitamins were skewed to the right, we performed a logarithmic transformation. In addition, we used multivariate linear regression models to evaluate the association between individual log-transformed vitamin intakes and obesity in children and adolescents.

Restricted cubic splines and correlation coefficient matrix plots were used to evaluate the non-linearity of multivitamins intake on obesity in children and adolescents and the interaction between vitamins. The BKMR analysis was performed to evaluate the combined effect of multi-vitamins based on Gaussian process regression. The BKMR method uses a Bayesian variable selection process to construct an exposure-response function and infer highly correlated vitamins. First, we analyzed the cumulative effect on obesity by fixing all nine vitamins to a specific percentile and increasing the 5th percentile. Then, while keeping the other eight vitamins at the median, we used the exposure-response function to explore the association between a single vitamin and obesity. Finally, the predicted response function of a single vitamin to other vitamins at different quantiles, and the rest of the vitamins fixed at the median was studied. The group posterior inclusion probability (groupPIP) and the conditional posterior inclusion probability (condPIP) were calculated, representing the probability of a mixture group and a particular vitamin within the group, respectively. In BKMR model, exposure variables with large PIP values may be important for the overall impact of obesity risk (25). BKMR model was fit 50,000 iterations using the Markov Chain Monte Carlo (MCMC) sampler.

Sensitivity analysis evaluates the robustness of the results by analyzing the correlation between dietary vitamin intake and metabolic parameters (including serum TC, serum HDL-C, serum LDL-C, fasting TAG and fasting plasma glucose concentrations, and HOMA-IR score).

The analysis was performed on the R software (version 3.6.0; R Core Team), and the statistical significance level on both sides was set to α = 0.05.

## Results

Our analysis included 3634 children and adolescents aged 6-19 from NHANES (2013- 2016). The median (IQR) age was 12 (9-16) years old, and the BMI was 20.80 (17.60-24.83) kg/m^2^ (**Table 1**). Among them, 533 children and adolescents were obese (14.7%). The majority of participants were non-Hispanic white (28%) and had an elementary school or lower education (52.8%). Their serum cotinine and daily energy intake were 0.03 (0.01-0.17) ng/mL and 1846 (1397-2392) kcal/d, respectively. The intakes of various vitamins were also presented in **Table 1**.

**Table 1:** Baseline characteristics among 3634 participants in the NHANES 2013–2016.

Characteristic	Frequency (%) or Median (IQR)
**Age (years)**	12 (9- 16)
**Gender**	
Female	1776 (48.9%)
Male	1858 (51.1%)
**Body mass index (kg/m^2^)**	20.80 (17.60- 24.83)
**Education level**	
Primary school and below	1920 (52.8%)
Middle school	855 (23.5%)
Senior high school and above	856 (23.6%)
**Race/ethnicity**	
Mexican American	845 (23.3%)
Other Hispanic	413 (11.4%)
Non-Hispanic White	1016 (28.0%)
Non-Hispanic Black	829 (22.8%)
Other/multi-racial	531 (14.6%)
**Poverty-income ratio**	1.29 (0.77- 2.94)
**Serum cotinine (ng/mL)**	0.03 (0.01- 0.17)
**Daily energy intake (kcal/day)**	1846 (1397- 2392)
**Vitamin A (mcg)**	475 (264- 765)
**Vitamin B_1_ (mg)**	1.44 (1.00- 2.02)
**Vitamin B_2_ (mg)**	1.74 (1.17- 2.45)
**Vitamin B_6_ (mg)**	1.54 (1.02- 2.25)
**Vitamin B_12_ (mcg)**	3.95 (2.23- 6.27)
**Vitamin C (mg)**	47.60 (18.40- 101.03)
**Vitamin D (mcg)**	3.90 (1.60- 7.10)
**Vitamin E (mg)**	6.30 (4.11- 9.43)
**Vitamin K (mcg)**	48.20 (28.80- 84.13)
**TC ≥ 200 mg/dl**	923 (25.3%)
**HDL-C ≤ 35 mg/dl**	318 (8.7%)
**LDL-C ≤ 130 mg/dl**	774 (21.2%)
**Fasting TAG ≥ 150 mg/dl**	691 (19.0%)
**Fasting glucose ≥ 100 mg/dl**	1506 (41.4%)
**HOMA-IR score ≥ 4.39**	797 (21.9%)

TC, total cholesterol; HDL-C, HDL-cholesterol; TAG, Triacylglyceride; LDL-C, LDL-cholesterol; HOMA-IR, homeostasis model assessment of insulin resistance.

In the multivariate logistic regression model (**Table 2**), we found that vitamin A, vitamin B_1_, vitamin B_2_, vitamin B_12_, and vitamin D were negatively associated with obesity in children and adolescents. The results of restricted cube plots and correlation coefficient matrix plots showed that multivitamins and obesity risk presented a non-linear correlation and there was a high correlation between vitamins (**Supplementary Figures 1, 2**).

**Table 2:** The association between each vitamin and body mass index in 3634 participants based on logistic regression model.

Vitamins	OR (95%CI)	P-value*
Vitamin E	0.96 (0.62-1.49)	0.853
Vitamin A	0.80 (0.63-0.99)	0.036
Vitamin B_1_	0.67 (0.41-0.84)	0.023
Vitamin B_2_	0.72 (0.48-0.93)	0.026
Vitamin B_6_	0.90 (0.61-1.34)	0.608
Vitamin B_12_	0.61 (0.39-0.81)	0.012
Vitamin C	0.98 (0.82-1.16)	0.770
Vitamin D	0.86 (0.60-0.96)	0.042
Vitamin K	0.84 (0.63-1.11)	0.211

*All models were adjusted for age, gender, race, education level, daily energy intake, serum cotinine, and poverty-income ratio.

Moreover, we used the BKMR model to evaluate the interaction between dietary intake of vitamins and obesity in children and adolescents. The estimated PIP of the BKMR model of vitamins and obesity risk in children and adolescents was shown in **Table 3**. The PIP of the three groups was higher than 0.5, and the condPIP of vitamin B_12_ (0.88) and vitamin D (0.65) were the highest in their groups.

**Table 3:** PIPs for group inclusion and conditional inclusion into obesity, using BKMR model.

Vitamin	Group	groupPIP	conPIP
Vitamin A	1	1	1
Vitamin E	4	0.32	0.16
Vitamin C	4	0.32	0.25
Vitamin D	4	0.32	0.41
Vitamin K	4	0.32	0.18
Vitamin B_1_	3	0.76	0.12
Vitamin B_2_	2	0.62	0.35
Vitamin B_12_	3	0.76	0.88
Vitamin D	2	0.62	0.65

PIPs, posterior inclusion probabilities; BKMR, Bayesian kernel machine regression. Model were adjusted for age, sex, education level, race/ethnicity, poverty-income ratio, serum cotinine and daily energy intake.

**Figure 2:**
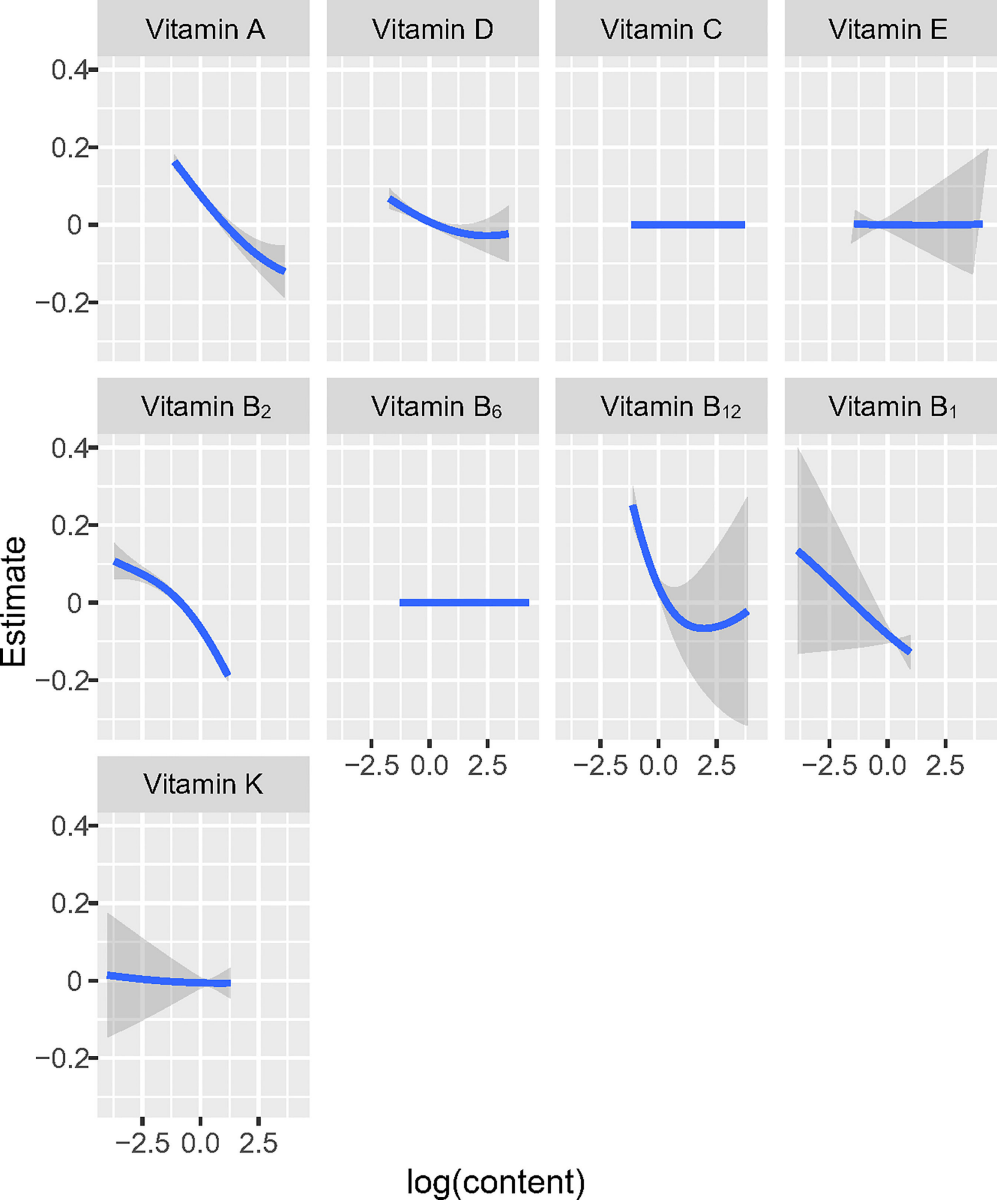
Univariate exposure-response functions and 95% confidence interval for association between the intake of single vitamin when the intake of other vitamins is fixed at the median.

At the same time, in order to investigate the potential nonlinearity of the exposure response function, the estimated univariate exposure-response function of each vitamin was demonstrated graphically in **Figure 2**. We found that the direction of exposure responses *via* the BKMR model was generally consistent with the associations observed in the single-vitamin multivariate logistic model. When all the other vitamins were fixed at their median concentrations, vitamin A, vitamin D, vitamin B_1_, vitamin B_2_ and vitamin B_12_ were positively correlated with high-density lipoprotein (HDL). The change in the content of the five vitamins from 25% to 75% was associated with a significant reduction in the risk of obesity.

**Figure 3:**
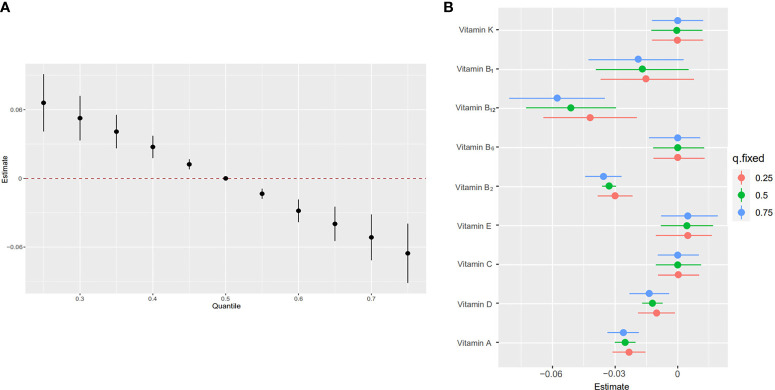
Joint effect [95% confidence intervals (CI)] of the five vitamins [vitamin A, vitamin B_1_, vitamin B_2_, vitamin B_6_, vitamin B_12_, vitamin C, vitamin D, vitamin E, vitamin K] on obesity by using Bayesian kernel machine regression (BKMR) model. The results were adjusted for age, sex, education level, race/ethnicity, poverty-income ratio, serum cotinine and daily energy intake. **(A)** Overall effect of five vitamins (estimates and 95%CI). The figure plots the estimated change in a latent continuous outcome (continuous marker of obesity) when all the vitamins at fixed percentiles were compared to all the vitamins at their 50th percentile. **(B)** Single vitamin association (estimates and 95% CI, estimated zero means null). This plot compares a latent continuous outcome when a single vitamin is at the 75th vs 25th percentile, when all the other vitamins are fixed at either the 25th, 50th, or 75th percentile.

**Figure 4:**
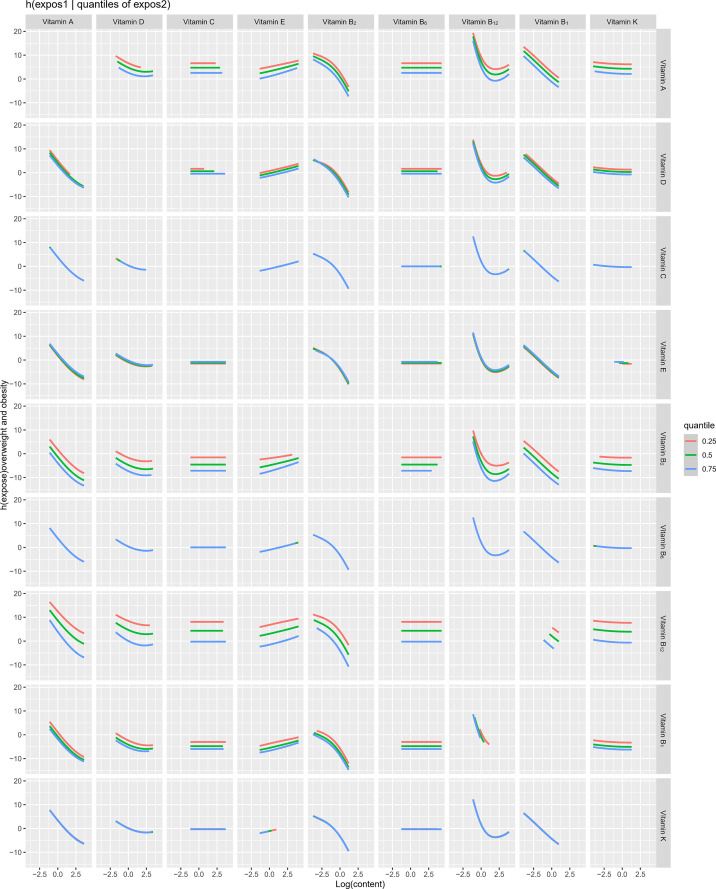
Bi-variate exposure response functions of each two vitamins in obesity among children and adolescents.

The overall associations between the vitamins mixture and obesity were shown in **Figure 3A**. The risk of obesity increased significantly with increasing total vitamins mixture levels. In order to assess the potential nonlinearity of the exposure-response function, we then estimated the univariate relationship between each vitamin in the pro-inflammatory nutrient group and the risk of obesity, with all remaining nutrients fixed at the 50th percentile. The risk of childhood obesity increased significantly with increased concentration levels of vitamin A, vitamin D, vitamin B_1_, vitamin B_2_, and vitamin B_12_ (**Figure 3B**). The **Figure 4** showed the interaction between vitamin B_2_ and vitamin B_12_ that may increase the risk of obesity in children and adolescents. When the other seven vitamins were at the median value, the positive slope of vitamin B_12_ became steeper at higher levels of vitamin B_2_.

The results of the sensitivity analysis showed that the associations between the nine dietary vitamins and metabolic parameters were consistent with the main results (**Supplementary Table 1**).

## Discussion

In this study, we found that the increase of five vitamins (vitamin A, vitamin B_1_, vitamin B_2_, vitamin B_12_, and vitamin D) was significantly negatively correlated with the occurrence of obesity in children and adolescents. The BKMR analysis showed that there was an interaction between vitamin B_2_ and vitamin B_12_. When the vitamin intake was higher than the 55th percentile, the mixed intake of the five vitamins had a negative effect on obesity in children and adolescents.

Vitamin A is essential in human nutrition because they can prevent certain diseases. Food intake and lifestyle could affect the serum concentration of this nutrient, especially fruits and vegetables containing vitamin A and its precursors are low in intake (26). Studies had shown that obese individuals were deficient in vitamin A (27, 28). However, it has not been determined whether this deficiency is due to poor diet or other factors, such as oxidative stress and inflammatory processes caused by obesity (29). In addition, studies had shown that BMI was negatively correlated with the serum concentrations of β-carotene, β-cryptoxanthin, retinol, and other carotenoids (α-carotene and lutein + zeaxanthin) (30, 31). Although subjects with different BMI ranges had similar carotenoid intakes, these studies showed that obese subjects had lower serum levels of vitamin A precursors. At the same time, studies had confirmed that higher serum levels of α-carotene, trans-β-carotene, and cis-β-carotene were associated with a lower risk of overweight or obesity in children, while an increase in retinol concentration was associated with an increased risk (32, 33). The mechanism that may lead to the discovery of an inverse association between serum carotenoids and obesity is the difference in dietary fruits, vegetables, and energy intake between obese and non-obese subjects (34). Although obese subjects may consume too much energy food, they may not be able to meet all their micronutrient needs. In addition, compared with lean people, obese/overweight individuals will deposit more b-carotene in their adipose tissue, so compared with lean people, the serum carotenoid concentration may be lower (7, 35).

Vitamin D deficiency is characterized by circulating 25-hydroxy vitamin D [25(OH)D] plasma levels below 20 ng/mL, which is associated with some chronic diseases, such as insulin resistance, metabolic syndrome, atherosclerosis and obesity (36, 37). Since the skin is exposed to the sun, the endogenous production of vitamin D is the main source of this vitamin, because very few foods contain this vitamin, and the content is at very low levels (38). The possible reason for the low vitamin D status during obesity is that obese individuals have low vitamin D intake, less physical activity, resulting in less exposure to the sun, and reduced intestinal absorption of nutritional vitamin D by individuals undergoing bariatric surgery (39–41). People living in high latitudes were rarely exposed to the sun, and the relationship between the low rate of endogenous vitamin D synthesis and the high rate of obese and overweight individuals had been confirmed (42). Increased obesity in children and adolescents is associated with vitamin D deficiency (43). Children with insufficient vitamin D intake (daily intake <70 IU) have higher body weight, BMI, waist circumference, and waist-to-hip (44). Contrary to other findings, adolescents showed the same relationship, but the concentration of parathyroid hormone did not increase (45). It seems that the relationship between vitamin D deficiency and obesity in adolescents is caused by metabolic factors in this age group, mainly due to hormonal changes (46, 47).

There is little evidence to support a direct causal relationship between vitamin D and obesity and metabolic parameters. Inflammation may be one of the mechanisms that explain the association between vitamin D and obesity and metabolic parameters (48). It has been reported that vitamin D can inhibit the concentration of 25(OH)D as an acute phase reactant in inflammation. Obesity is now widely regarded as a chronic, low-grade systemic inflammatory state (49, 50). It has been reported that the improvement of 25(OH)D concentration can enhance the beneficial effect of weight loss, which may be at least partly attributed to the anti-inflammatory effect of vitamin D. Although a meta-analysis of randomized clinical trials had shown that vitamin D supplementation had no effect on inflammatory biomarkers in overweight/obese adults, more research evidence is needed. Recently, studies had shown that vitamin D supplementation could reduce inflammation biomarkers [ie interleukin-6 (IL-6) levels] in adults with metabolic syndrome. More randomized clinical trials in obese children and adolescents are needed to clarify the mechanism (51, 52). At the same time, obesity-related vitamin D deficiency may be due to the reduced bioavailability of subcutaneous fat tissue and diet-derived vitamin D, because it is deposited in various fat areas of the body. In addition, LC-MS/MS detected vitamin D3 in subcutaneous adipose tissue of obese subjects, and the results showed that the concentration of vitamin D3 in adipose tissue was more than 10 times higher than that in serum. These findings indicate that vitamin D, as a fat-soluble vitamin, may accumulate and segregate in adipose tissue and cannot enter the circulation to produce 25(OH)D in the liver. This may lead to a decrease in plasma 25(OH)D levels in subjects with excessive accumulation of adipose tissue (53, 54).

Few comparable studies had investigated the relationship between children’s vitamin B intake and physical obesity. A study reported that the intake of niacin in overweight boys was significantly lower than that of non-overweight boys (55). Studies had found that long-term use of vitamin B-6 and B-12 supplements is significantly associated with lower levels of weight gain in overweight or obese men and women (56, 57). These studies, combined with the findings of B-vitamins status and obesity, strengthen the argument that it may play an important role in adipogenesis. This association may be caused by the inflammatory state found in obese individuals, which can lead to changes in the physiology of the B-vitamins (58). Obesity can induce systemic oxidative stress, which may lead to imbalance of adipocytokines and systemic inflammation, and subsequently reduces the serum concentration of vitamin B-6 (59). For example, A study had found that low-circulation 5’-pyridoxal phosphate (PLP) was associated with an increase in inflammation marker C-reactive protein. It had also been found that there was a negative correlation between dietary intake of PLP or vitamin B-6 and coronary heart disease (60). These findings indicated that when there was an underlying inflammatory process, the use of vitamin B-6 could decrease the concentration of PLP in obesity and coronary heart disease.

A new BKMR analysis method was implemented to capture the reliable association between environmental multivitamins intake and obesity in children and adolescents. We found that a mixture of vitamin A, vitamin B_1_, vitamin B_2_, vitamin B_12_, and vitamin D had a significant positive combined effect on the risk of obesity in children. Therefore, any potential intervention for vitamin intake imbalances should specifically consider multivitamins instead of a single vitamin (61, 62). The BKMR results provided a non-linear analysis of the interaction between vitamins and a significant positive interaction between vitamin B_2_ and vitamin B_12_ was observed in this study. The mechanism underlying the observed interactions between B-vitamins and changes in BMI remains unknown. However, the current findings are biologically reasonable, because B-vitamins play an important role in the DNA methylation of obesity-related genes (63). In addition, vitamin B_2_ and vitamin B_12_ are the main determinants of one-carbon metabolism, among which methyl donors are formed. It had been studied that the co-expression of the Vitamin B_2_ and vitamin B_12_ could increase the risk of obesity (64, 65).

The strengths of our research included a large sample size and sufficient statistical power to evaluate potential interactions. In addition, we used BKMR to explain the potential interaction between vitamin mixtures and obesity in children and adolescents. The most important limitation of the study was the cross-sectional design, which limited our ability to assess causality. In addition, we only evaluated the dietary intake of vitamins, which may overestimate the impact of vitamin content on obesity in children and adolescents. At last, a 24-hour recall may not be the best tool for assessing daily intake. For vitamins that vary greatly from day to day due to their distribution in different foods, this may introduce potential biases to the results.

## Conclusion

We discover a combined negative effect of multivitamins on obesity in children and adolescents, and observe a significant interaction between vitamin B2 and vitamin B12. Further cohort studies are needed to clarify the health effects of multivitamins intake in larger population.

## Data Availability Statement

The original contributions presented in the study are included in the article/**Supplementary Material**. Further inquiries can be directed to the corresponding author.

## Ethics Statement 

The studies involving human participants were reviewed and approved by the National Center for Health Statistics Ethics Review Board. Written informed consent to participate in this study was provided by the participants’ legal guardian/next of kin.

## Author Contributions

QC: design, analysis, drafting and revising manuscript. WT: design, analysis, drafting and revising manuscript. WZ: design, analysis and revising manuscript. MW: data collection and revising manuscript. All authors contributed to the article and approved the submitted version.

## Funding

The project was supported in part by National Natural Science Foundation of China (NSFC 81803244) and Collaborative Innovation Program of Shanghai Municipal Health Commission (2020CXJQ01). The funders did not play any role in the study design, data collection and analysis, decision to publish or preparation of the manuscript.

## Conflict of Interest

The authors declare that the research was conducted in the absence of any commercial or financial relationships that could be construed as a potential conflict of interest.

## Publisher’s Note

All claims expressed in this article are solely those of the authors and do not necessarily represent those of their affiliated organizations, or those of the publisher, the editors and the reviewers. Any product that may be evaluated in this article, or claim that may be made by its manufacturer, is not guaranteed or endorsed by the publisher.
