# Supplementary material for: Associations Between Different Dietary Vitamins and the Risk of Obesity in Children and Adolescents: A Machine Learning Approach
Source: Front Endocrinol (Lausanne). 2022 Feb 17;12:816975. doi: 10.3389/fendo.2021.816975 (PMC8893992; doi:10.3389/fendo.2021.816975)
Supplement: Supplementary file 1 [file DataSheet_1.docx]

**Supplementary materials**

**
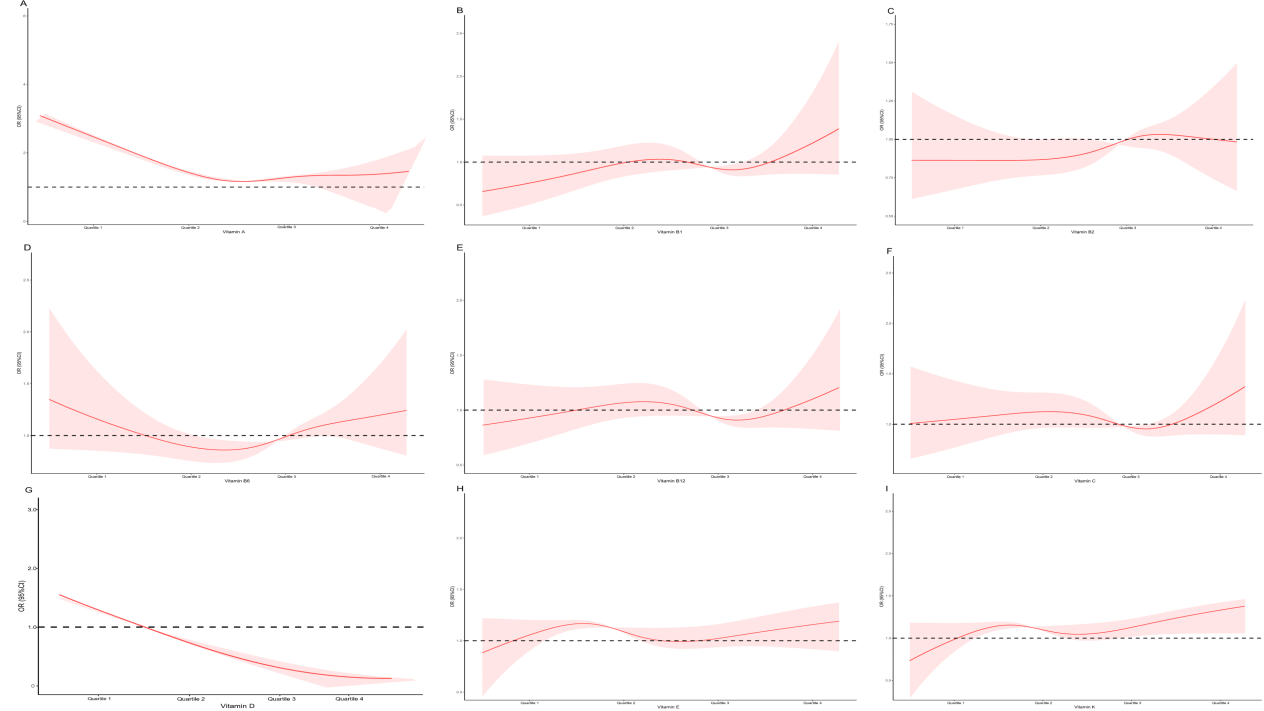
**

**Supplementary Figure 1** Restricted cubic spline of the nonlinear relationship between obesity risk in children and adolescents and increased vitamin concentration. (A),Vitamin A;(B),Vitamin B1;(C),Vitamin B2;(D),Vitamin B6;(E)Vitamin B12;(F),Vitamin C;(G),Vitamin D;(H),Vitamin E;(I),Vitamin K. The solid line displays the odds ratio (OR), and the dashed line represents the 95% confdence interval (CI).

**
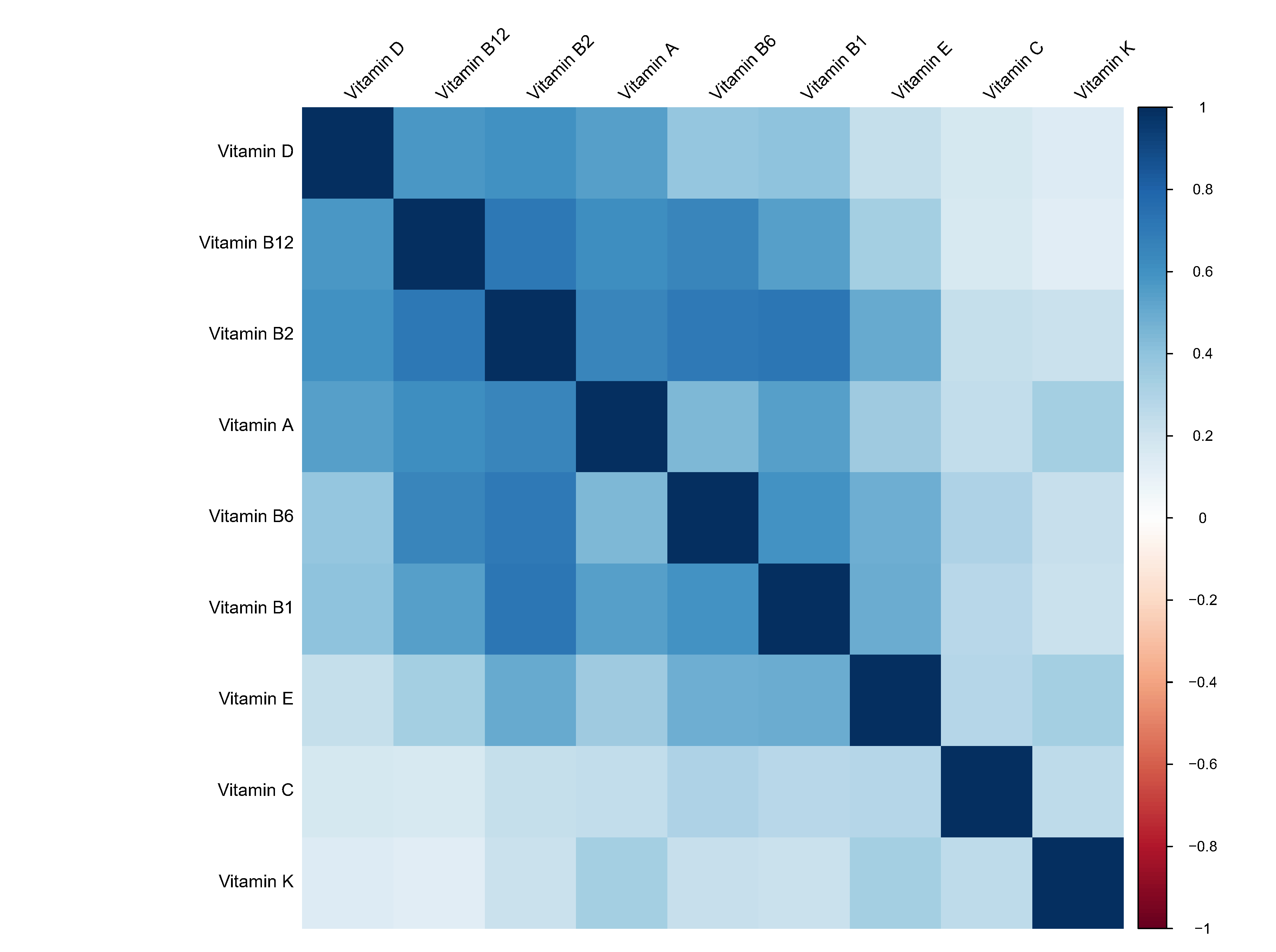
**

**Supplementary Figure 2** Matrix graph of the correlation coefficient between vitamins.
